# Analytical markers for silk degradation: comparing historic silk and silk artificially aged in different environments

**DOI:** 10.1007/s00216-014-8361-z

**Published:** 2014-12-10

**Authors:** Francisco Vilaplana, Johanna Nilsson, Dorte V. P. Sommer, Sigbritt Karlsson

**Affiliations:** 1 Division of Glycoscience, School of Biotechnology, AlbaNova University Centre, KTH Royal Institute of Technology, 106 91 Stockholm, Sweden; 2 Department of Fibre and Polymer Technology, School of Chemical Science and Engineering, KTH Royal Institute of Technology, Teknikringen 46-48, 100 44 Stockholm, Sweden; 3 Department of Collections, The Royal Armoury, Skokloster Castle, Hallwyl Museum, Slottsbacken 3, 111 30 Stockholm, Sweden; 4 School of Conservation, Royal Danish Academy of Fine Arts Schools of Architecture, Design and Conservation, Esplanaden 34, 1263 Copenhagen, Denmark; 5 University of Skövde, Box 408, SE-541 28 Skövde, Sweden

**Keywords:** Silk, Conservation, Multivariate analysis, Amino acid composition, Infrared spectroscopy, Mechanical properties

## Abstract

Suitable analytical markers to assess the degree of degradation of historic silk textiles at molecular and macroscopic levels have been identified and compared with silk textiles aged artificially in different environments, namely (i) ultraviolet (UV) exposure, (ii) thermo-oxidation, (iii) controlled humidity and (iv) pH. The changes at the molecular level in the amino acid composition, the formation of oxidative moieties, crystallinity and molecular weight correlate well with the changes in the macroscopic properties such as brightness, pH and mechanical properties. These analytical markers are useful to understand the degradation mechanisms that silk textiles undergo under different degradation environments, involving oxidation processes, hydrolysis, chain scission and physical arrangements. Thermo-oxidation at high temperatures proves to be the accelerated ageing procedure producing silk samples that most resembled the degree of degradation of early seventeenth-century silk. These analytical markers will be valuable to support the textile conservation tasks currently being performed in museums to preserve our heritage.

**Electronic supplementary material:**

The online version of this article (doi:10.1007/s00216-014-8361-z) contains supplementary material, which is available to authorized users.

## Introduction

Silk from the cultivated *Bombyx mori* larva is an appreciated material in historic costumes due to its valuable properties such as high lustre, smoothness, strength and lightness. However, silk is one of the natural fibres most sensitive to environmental degradation factors that cause deterioration of its intrinsic properties, becoming fragile and therefore difficult to preserve. The Royal Armoury in Stockholm possesses extraordinary costumes in silk from the seventeenth century, both in quantity and quality, which are in need of conservation. Conservators concerned with the preservation of historic silk must assess the degradation level in the material when deciding whether an item can be exhibited and how it should be treated for future preservation. It is therefore necessary to understand the environmental degradation mechanisms that affect silk through their ageing and to find reliable analytical markers for monitoring the degree of degradation of historic silk textiles.

Silk is a highly oriented and crystalline proteinaceous fibre containing mainly fibroin and sericin proteins. It consists of a double filament of fibroin with sericin acting as glue around the two filaments. The filament is 7–12 μm in width and composed of fibrillar elements of 1 μm width, in turn made up of microfibrils that are 10 nm in diameter [1]. Silk fibroin consists of two main components, H-fibroin with molecular weight of about 350,000 Da and a smaller L-fibroin with about 25,000 Da, which are linked by a single intermolecular disulphide bridge [2]. A third component is the glycoprotein P25, which is present in a much smaller proportion than the two others, and associated with the H- and L-fibroins by non-covalent forces [3, 4]. The molar ratio between H-fibroin, L-fibroin and P25 is 6:6:1 [5]. The H-fibroin primary structure consists of 20 amino acids, mainly glycine (Gly) 45.9 %, alanine (Ala) 30.3 %, serine (Ser) 12.1 % and tyrosine (Tyr) 5.3 %. The amino acid sequence consists of alternating crystalline and amorphous subdomains. The crystalline domains are made of a repeated Gly-X dipeptide motif, where X is Ala in 65 %, Ser in 23 % and Tyr in 9 % of the cases [6]. Gly-X dipeptide units are present mainly as part of the two hexapeptides ~Gly-Ala-Gly-Ala-Gly-Ser~ (433 copies) and ~Gly-Ala-Gly-Ala-Gly-Tyr~ (120 copies) which together count for 70 % of the crystalline domains. The Gly-X repeats are distributed in 12 “crystalline” domains with varying length between 39 and 612 amino residues, separated by almost identical copies of boundary “amorphous” sequences. These amorphous spacers in *B. mori* silk are tyrosine rich and also contain most of the other amino residues that are absent in the Gly-X domains, basically amino acids with bulky and polar side chains. These amorphous domains break the Gly-X alternate and terminate the crystalline regions. The Gly-X alternation is strict within the crystalline subdomains; this strongly supports the classic-pleated β-sheet model of secondary structure, in which β-sheets pack on each other in alternating layers of Gly/Gly and X/X contacts. In the amorphous regions, however, distorted β-sheets are present [7].

Research on environmental factors influencing silk objects in historic houses is usually performed by accelerated ageing test methods [8–10]. Exposure to light is usually thought to play an important role in changing historic silk’s chemical and physical properties as well as its aesthetics. However, previous studies strongly suggest that the role of light on the deterioration of historic silk textiles may be exaggerated [11]. Ultraviolet (UV) irradiation is a common procedure to artificially mimic the effect of daylight on historic silk textiles; however, accelerated UV irradiation affects the structure and the properties of silk textiles in a different way than natural daylight exposure, resulting in crosslinking of the material and altered mechanical properties [12]. Extreme humidity conditions, both at high and low levels, are known to have negative effects on silk by aiding deterioration. Moreover, temperature affects and accelerates deterioration, reduces molecular weight and has a negative effect on tensile strength [8–14]. In general, these previous studies use different artificial ageing procedures to analyze the chemical structure and properties of silk fibroin in comparison with historic samples, but no comprehensive correlation between the mechanical properties and the molecular structural details is proposed. The aim of the present research is to identify suitable analytical markers at the molecular and macroscopic level and establish correlations amongst them, in order to assess the degree of degradation of historic silk. The changes at the molecular level (amino acid composition, formation of oxidative moieties, crystallinity and molecular weight) and on the macroscopic properties (pH, brightness, mechanical properties) have been investigated and integrated using statistical approaches. Analytical results of three historic samples from the seventeenth century have been correlated with artificially aged silk in four different environments, namely (i) UV exposure, (ii) thermo-oxidation, (iii) controlled humidity and (iv) pH. Significant and extensive data has been obtained for the first time about the tensile properties (elongation at break, tenacity, modulus) of both artificially aged and historic silk textiles. These analytical markers are used to better understand the degradation mechanisms that silk textiles undergo during prolonged exposure and to support the heritage preservation tasks currently performed in our museums.

## Experimental

### Materials

**Fig. 1:**
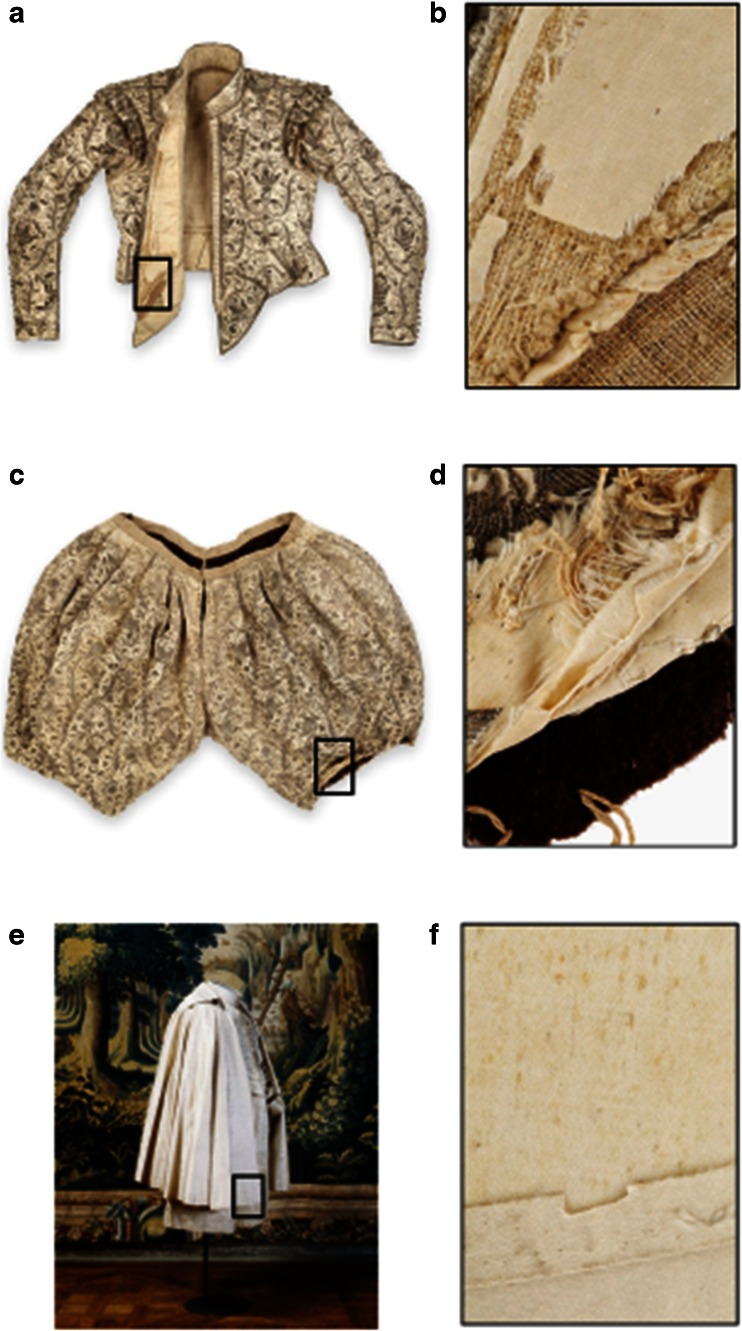
Historic silk costumes and sampling procedure. **a** King Gustav II Adolf (GIIA) doublet showing where sample was taken from the lining; **b** detail of GIIA doublet lining; **c** GIIA breeches showing where sample was taken; **d** detail of the seam allowance. **e** King Karl X Gustav (KXG) cloak showing where samples were taken; **f** detail of seam allowance. Photo: **a**–**d**, **f** courtesy of Erik Lernestål; **e** courtesy of Göran Schmidt

White reference silk fabric, ISO 105-F06:2000 *B. mori* (Cromocol, Boras, Sweden) was used for artificial ageing. The silk has an average weight of 60 g m^−2^ and a defined tone and grade of whiteness and pH according to ISO 3071. The manufacturing process of the historic silk differs from the one used today for modern standard silk, but for this study, it was not possible to make exact copies produced in the same way as historic silk. The historic silk samples were taken from the costume collection at the Royal Armoury (Stockholm), specifically from the doublet (inventory number Lrk 25605) and from the breeches (inventory number Lrk 25606) of the coronation costume of King Gustav II Adolf (GIIA) from 1617 and from the coronation cloak (inventory number Lrk 25599) of King Karl X Gustav (KXG) from 1654. Samples were taken from the weave in the seam allowance taking special care to avoid external contamination (Fig. 1). The chemicals and buffers were supplied by Sigma-Aldrich, USA.

### Accelerated ageing of silk

Samples of reference silk were artificially aged in groups of five replicates by exposure to four different environmental conditions: (a) accelerated UV exposure for 1–10 days; (b) thermo-oxidation in dry air at 60 and 125 °C for 14, 21, 28, 35, 42, 49 and 56 days; (c) exposure to 0, 53, 75, 86 and 100 % relative humidity (RH) at 25 and 60 °C for 28 days; and (d) immersion in and exposure to solutions of pH 1, 3, 7, 11 and 13 at 25 °C and at 60 °C for 28 days. Exposure to a controlled temperature in methods b–d was carried out using a forced ventilation oven, Memmert D06062 (Memmert Gmbh, Germany). The samples subjected to method b–d; sample size 5 × 8 cm) were mounted in sealed vials. The relative humidity was controlled by saturated salt solutions: dry calcium chloride (CaCl_2_) was used for 0 % RH, saturated solutions of magnesium nitrate (Mg(NO_3_)_2_) for 53 % RH, sodium chloride (NaCl) for 75 % RH, potassium chloride (KCl) for 86 % RH and distilled water for 100 % RH. Different buffers from Sigma-Aldrich were used to control pH exposure: Hydrogen chloride/potassium chloride (HCl/KCl) was used for pH 1, citric acid/sodium hydroxide/sodium chloride buffer (C_6_H_8_O-/NaOH/NaCl) for pH 3, potassium phosphate/disodium hydrogen phosphate buffer (KH_2_PO_4_/Na_2_ HPO_4_) for pH 7, boric acid/sodium hydroxide/potassium chloride buffer (B(OH)_3_/NaOH/KCl) for pH 11, sodium chloride/glycine/sodium hydroxide buffer (NaCl/glycine NaOH) for pH 13. Samples were either immersed or exposed over the pH buffer to control the effect of the pH degradation. Accelerated UV exposure was performed in a Q-U-V Accelerated Weather Tester with a UVB 313 lamp according to ASTM G 53–96, with a peak irradiance at 313 nm and total irradiance of 0.5 W.m^−2^ at 50 ± 2 °C at 95 % RH for 1–10 days in a cycle of 8 hours exposure and 4 hours condensation at a temperature of 50 ± 2 °C. The samples for UV exposure were cut with dimensions 7.5 × 15 cm. After exposure to the different degradation environments, the samples were rinsed five times in deionised water and dried at room temperature at ambient humidity in a covered glass box (80 dm^3^) for 24 h. Samples were kept in desiccators prior to analysis.

### Analysis of silk pH

The method to determine pH was miniaturised following the guidelines of previous studies [10, 15, 16]; 12 mg silk was soaked in 1.0 ml degassed 100 mM NaCl solution at 20 ± 2 °C for 30 min until equilibration and the pH of the extract was measured with a BDH Glass + combination microelectrode, following two point calibration (pH 4.00 and 7.00). Depending on sample availability, the pH was determined for selected samples including the unaged reference standard silk, UV exposed for 4 and 10 days, dry thermal-oxidation at 125 °C in 28 and 56 days, immersed in solutions of pH 1 or pH 13 at 25 °C in 28 days, 100 % humidity at 25 °C in 28 days and samples from the three historic costumes.

### Brightness measurements

The sample brightness was measured in triplicate on the artificially aged silk textile samples using a UV–vis spectrophotometer equipped with an integrating sphere (Varian) and reported as the transmittance values (%) at 457 nm. The brightness could unfortunately not be measured for the historic silk textiles due to the large sample size required for such measurements.

### Fourier-transform infrared spectroscopy

The chemical changes on the surface of the silk samples were monitored by FTIR using a Spectrum 2000 FTIR spectrometer (Perkin Elmer, Wellesley, MA) equipped with attenuated total reflection (ATR). Spectra were collected from the average of 24 scans between 2000 and 600 cm^−1^ at intervals of 1 cm^−1^ with a resolution of 4 cm^−1^. The spectral data for each sample was subjected to baseline correction and to normalisation towards the absorption at 1800 cm^−1^, where no absorbance band was observed in any of the samples, using the Spectrum FTIR software. Each measurement was performed in triplicate, and the quantitative results for the carbonyl and amide II crystalline indexes were calculated as the averages from these measurements.

### Amino acid composition

Between 80 and 145 μg of silk sample were hydrolyzed for 24 h in an evacuated and sealed glass ampoule at 110 °C in a solution of 100 μl 6 M redistilled HCl, 5 μl 1 % 3,3′-dithiodipropionic acid (DTDPA) in 0.2 M NaOH and 5 μl 1 % phenol in water. The hydrolyzed amino acids were separated by ion exchange HPLC (Waters, USA) with two Waters high pressure pumps, equipped with high sensitivity pulse dampers and microflow modules, Waters M 717, refrigerated auto sampler, two Reagent Manager pumps and a column oven. Separation was carried out in a 15 × 0.40 cm steel column packed with MCI CK 10 U resin (Mitsubishi Chemical Industries) using a pH gradient system with two buffers: (A) pH 3.10:0.20 sodium citrate containing 0.65 % nitric acid and 5 % isopropanol; (B) pH 10.20:0.210 M sodium borate, 5 % isopropanol and 0.17 M sodium hydroxide. The eluted amino acids were quantified by post-derivatisation with ortho-phthalaldialdehyde (OPA) using a Waters M 474 fluorescence detector with 338 nm band-pass excitation filter and 450 nm long-pass emission filter. The amino acids were identified and quantified on the basis of an external standard mixture of amino acids Beckman no 33 1018, with additional Hydroxylysine and the degradation products α-aminoadipic acid (α-Ada), α-aminobuteric acid (α-Abu), β-alanine (β-Ala), γ-aminobuteric acid (γ-Abu), 6-aminohexanoic acid (6-Aha) and ornithine (Orn). The results are compared with a model silk sequence (MSS) calculated from the amino acid sequences of the *B. mori* fibroin heavy chain (sequence number P05790, UniProtKB/Swiss-Prot), *B. mori* fibroin light chain (sequence number P21828, UniProtKB/Swiss-Prot) and the sequence of fibroin P25 (sequence number P04148, UniProtKB/Swiss-Prot). The reported values are the sum of the three in the ratio 6:6:1 [11]. The amino acid composition was determined for selected silk samples (same as for the pH measurements).

### Size-exclusion chromatography

Size-exclusion chromatography (SEC) was performed in a LC-20AD liquid chromatography instrument with refractive index detection (RID; Shimadzu, Japan). Between 3 and 8 mg of sample were initially washed at room temperature in water (3 × 30 min) and methanol (3 ×3 0 min) to swell and open up the structure. After filtering, 1 ml *N*,*N*-dimethylacetamide (DMAc) with 8 % (*w*/*w*) lithium chloride (LiCl) was added to the silk and left under agitation at room temperature until the silk was completely dissolved. The silk solution was further diluted to achieve injection conditions (DMAc/0.5 % (*w*/*w*) LiCl). Separation was performed using a PL gel 20 mm Mixed-A column (Agilent, USA) at 40 °C using DMAc/0.5 % (*w*/*w*) LiCl as a mobile phase with a flow of 0.5 ml min^−1^. Relative calibration was performed using Pullulan standards (Agilent, USA).

### Tensile tests

The tensile tests were conducted in a Vibrodyn ® CRE-type with a gauge length of 20 mm, a tension weight of 2000 mg and an extension rate of 20 mm/min, following DIN EN ISO 5079 standard*. Samples were conditioned at 20 ± 2 °C and 65 ± 3 % RH prior to analysis. Eight 40-mm threads taken from the weft of each historic costume were tested; they were taken in the seam allowance of the breeches and the cloak and in a damaged area of the doublet. All extracted threads had almost no twist. Analyses of the historic silk showed that it was degummed but had small residues of sericin. A minimum of five threads from each artificially aged sample were removed from the weft, their *tex* value was calculated by measuring the length and weight of each thread and were finally subjected to tensile testing. The tensile tenacity (cN/*tex*), break extension (%) and Young’s modulus were obtained as the average from the tensile testing. Tests of significance, one-way ANOVA, were performed to ensure that reliable effects were obtained. The focus was first on general different effects of the exposures to the ageing methods, followed by tests of pair-wise differences where unaged reference silk was one part if the general difference was found to be significant. When appropriate, Welch’s method and Tamhane’s T were applied. In the studies of the effect of RH that had two sources of variance, univariate ANOVA was used to estimate the individual effect on variance caused by different sources as well as their interaction (SPSS Statistics, version 19).

## Results and discussion

### pH measurements

**Fig. 2:**
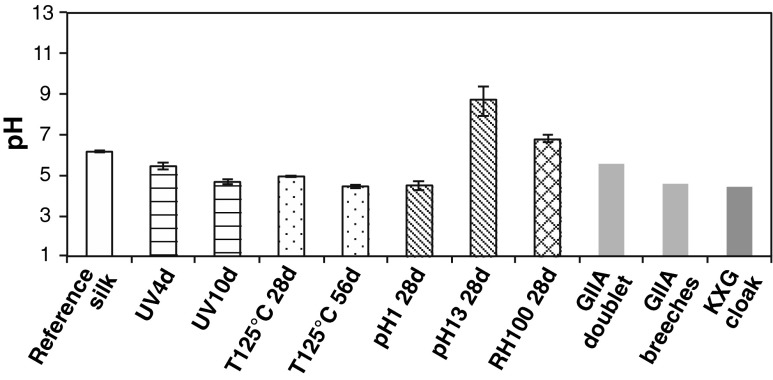
Extract acidity of the historic and artificially aged silk textiles

Acidity is a traditional indicator of silk degradation in textile conservation science. Low pH values during textile washing are usually correlated with specimen deterioration and this needs to be taken into account during their conservation to avoid further catalytic degradation action. Therefore, a neutral pH is strived for during textile preservation. In general, accelerated aged silk shows lower pH values than the reference silk and the acidity increased progressively with exposure time for the UV and thermo-oxidised samples (Fig. 2). The historic samples show similar pH values to the samples exposed to accelerated UV for 10 days and the samples subjected to thermal-oxidation at 125 °C for 28 days. This increased acidity indicates changes in the chemical structure of the silk fibres with natural and artificial ageing that will be studied in detail using integrated physico-chemical analyses.

### Brightness measurements

**Fig. 3:**
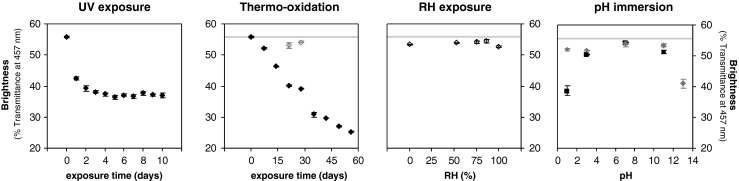
Effect of the different artificial degradation environments on the brightness (expressed as % of transmittance at 457 nm) of silk textiles. **a** UV exposure; **b** thermo-oxidation (*black*, 125 °C; *grey* 60 °C); **c** RH exposure (*black*, 60 °C); **d** pH immersion (*black square* 60 °C; *grey rhombus*, 25 °C). The *area in grey* corresponds with the reference silk

Brightness is a commonly used visual degradation marker that is related to colour changes (yellowing) in non-dyed silk textiles. The brightness results from the artificially aged silk exposed to different environments are presented in Fig. 3 and in the Electronic supplementary material (ESM Fig. S1). UV irradiation causes a marked exponential initial decrease in brightness, which stabilises after 2–4 days. Contrarily, thermo-oxidation at high temperatures (125 °C) causes a progressive linear decrease in brightness with exposure time, whereas lower exposure temperatures do not cause such dramatic reduction in brightness. Indeed, after 28 days of exposure to thermo-oxidation at 60 °C, no remarkable changes in brightness could be observed. Exposure to relative humidity at 25 and 60 °C for 28 days does not affect the brightness of the silk samples. Finally, only immersion into extreme acid pH solutions (both acidic at pH 1 and alkaline at pH 13) causes a dramatic decrease in brightness, similar to that of UV exposure. Milder alkaline and acidic pH conditions do not cause a marked alteration in the brightness after immersion or exposure for 28 days at neither 25 nor 60 °C. The sample immersed at 60 °C at pH 13 could not be measured since it completely disintegrated during exposure. The decrease in brightness is thought to be caused by the formation of chromophoric groups (e.g. oxidative moieties) during the degradation environments to which silk textiles are exposed; this will be correlated with the chemical changes by FTIR and with the amino acid content.

### Fourier-transform infrared spectroscopy

**Fig. 4:**
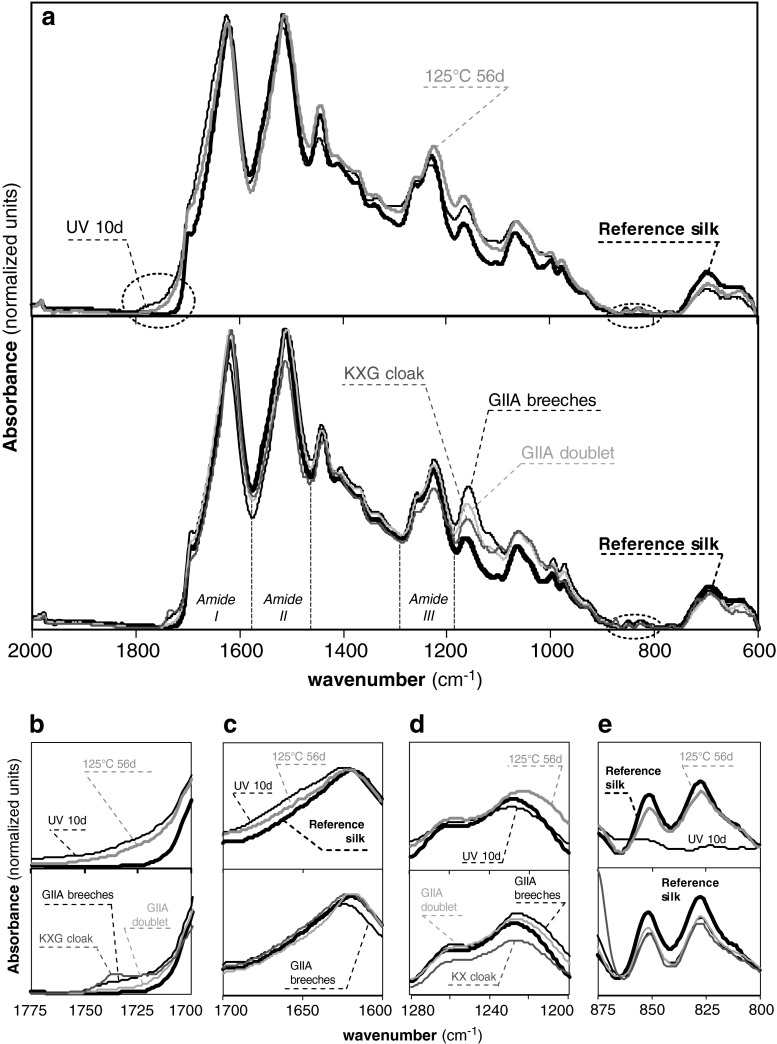
FTIR spectra of selected artificially aged and historic silk textiles: **a** overview of the mid-infrared region (600–2000 cm^−1^); **b** free carbonyl region (1700–1775 cm^−1^); **c** amide I region (1600–1700 cm^−1^); **d** amide III region (1200–1280 cm^−1^); tyrosine doublet region (800–875 cm^−1^)

Chemical and structural changes were monitored using FTIR for the accelerated aged and historic silk samples. Figure 4a shows selected spectra in the region between 600 and 2000 cm^−1^, where the main bands corresponding to the amide I (1580–1700 cm^−1^), amide II (1480–1580 cm^−1^), amide III (1200–1300 cm^−1^), C–H bending (1175 cm^−1^) and skeletal stretching (900–1100 cm^−1^) regions can be observed [17, 18]. The FTIR spectra for the rest of the accelerated aged silk samples were also obtained but are not presented here for the sake of clarity. These major bands correspond mainly to the associated backbone vibrations of the peptide chains in the Gly-Ala-Gly-Ala-Gly-X sequence (X accounts for Ser or Tyr), which accounts for the vast majority of the crystalline domains in silk fibroin and also correspond with above 90 % of the amino acid content in our studied silk textiles (see “Amino acid composition”). Two small bands can be observed at wavenumbers of 835 and 850 cm^−1^, which are assigned to the phenyl groups of Tyr.

**Fig. 5:**
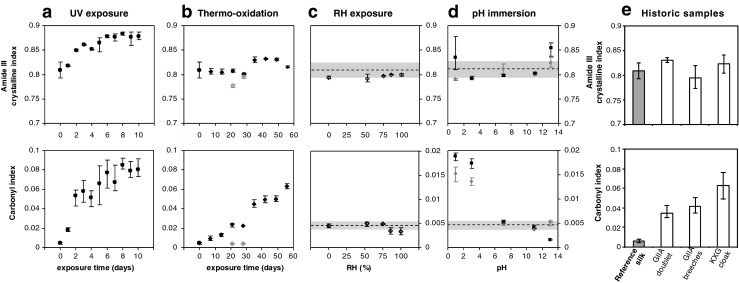
FTIR crystalline and carbonyl indexes: **a** UV exposure; **b** thermo-oxidation (*black*, 125 °C; *grey*, 60 °C); **c** RH exposure (*black*, 60 °C); **d** pH immersion (*black squares*: 60 °C; *grey rhombus*, 25 °C); **e** historic silk samples. The *area in grey* corresponds with the reference silk

The most important differences in the spectra are the appearance of absorbing bands in the free carbonyl region (1700–1775 cm^−1^) for both the accelerated aged and the historic costumes when compared with the standard silk (Fig. 4b). The formation of free carbonyl moieties is a common indicator of oxidation processes in natural and synthetic macromolecules, and it can be used to evaluate the extent of degradation processes. At a first glance, UV and thermo-oxidative exposure at 125 °C cause the presence of such carbonyl bands, whereas pH exposure and RH do not cause such effect. Historic costumes do exhibit absorption in the carbonyl region, which evidences the extent of oxidation in such textiles. The formation of carbonyl moieties caused by natural and accelerated degradation can be quantified using the carbonyl index. The carbonyl index was calculated from the height intensities at a wavenumber of 1732 cm^−1^ corresponding to the C=O vibration, normalised to the maximum height of the amide I band at 1620 cm^−1^. We verified the sensitivity of the amide I band towards degradation by evaluating the height of the maximum of the band at 1620 cm^−1^ for all the artificially aged and historical samples, and no marked changes were observed for any of the degradation environments or the historic samples (ESM Fig. S2), even though peptide hydrolysis occurred in certain conditions. Other studies on degradation in archaeological scrolls have observed an increase in the absorbance at 1650 cm^−1^ relative to OH– bending in the amide I region due to major hydrolysis of the collagen protein present in the parchments [19]. Collagen is also a fibrous protein, but unlike silk, collagen forms triple α-helix structures that may be more susceptible to hydrolytic degradation than the β-sheet secondary structures in silk fibroin. The carbonyl index for the artificially aged samples (UV radiation, thermo-oxidation, RH and pH) and the historic costumes are presented in Fig. 5 and ESM Fig. S3. UV exposure causes a drastic increase in the carbonyl index, especially for the shorter exposure times, tending to stabilise for longer times (Fig. 5). Thermo-oxidative exposure at lower temperatures (60 °C) does not cause an increase in the carbonyl index after 28 days. Higher temperatures (125 °C), on the other hand, cause a progressive increase in the carbonyl groups, reaching at 60 days similar values to those exhibited after UV radiation (Fig. 5). RH exposure does not induce changes in the carbonyl region after 28 days, neither at ambient temperature (25 °C) (ESM Fig. S3) nor at higher exposure temperatures (60 °C) (Fig. 5). Immersion into aqueous solutions with extreme acidic pH conditions (pH 1 and pH 3) for 28 days causes a marked increase in the carbonyl index, especially at higher temperatures of 60 °C but also at ambient temperatures (Fig. 5). Exposure to environments with extreme acidic pH conditions does not cause such marked effect as direct immersion (ESM Fig. S3). The effect of pH immersion on the carbonyl region is however lower than the changes caused by UV and thermo-oxidative exposure. Extreme acidic conditions may catalyse oxidative reactions undergone by the exposed or immersed silk that arise to the formation of carbonyl groups. On the other hand, extreme alkaline conditions do not induce any alteration of the carbonyl region. The results for historic silk samples indicate that oxidative reactions have indeed occurred during the natural ageing of the textiles. The carbonyl index of the samples from the seventeenth century is at the same level as the thermo-oxidised silk at 125 °C (Fig. 5). These results evidence the importance of the carbonyl index as a fundamental analytical marker to monitor the degradation state of historic silk samples. This could lead to the mapping of the degree of degradation of historic objects as a tool in collection management.

An interesting feature of this study is that neither artificial nor natural ageing affect to a great extent the features of the main band corresponding to amide I (Fig. 4c). The amide I band encodes information about protein conformation through the overlapping of the signals corresponding to α-helix, β-sheet and amorphous peptide chains [17]. The amide I region for standard and aged silk samples exhibits a main band at 1615 cm^−1^ corresponding to hydrogen bonding between intermolecular β-sheet strands, a broad band at around 1650–1660 cm^−1^ for random coil chains and an elbow at 1690 cm^−1^ assigned to β-turns of the antiparallel β-sheet structure [20–22]. The comparison of the FTIR signals from the amide I region indicates that the silk fibres do not undergo any major conformational transition induced by the natural or accelerated degradation processes. This observation reinforces the use of the amide I band as a reference for the calculation of the carbonyl index.

For a clearer observation of the secondary conformation of the aged silk samples from *B. mori*, the region corresponding to the amide III was analysed in detail (Fig. 4d). Two distinct bands can be observed at 1227 and 1265 cm^−1^, which can be assigned to the random coil and β-sheet configurations, respectively [18]. In principle, no clear changes in the conformation can be observed after accelerated or natural ageing, in agreement with the previously reported results from amide I. However, a more detailed analysis by calculating the amide III crystalline index using the relative height intensities from the bands at wavenumbers of 1260 and 1227 cm^−1^ reveals subtle changes in the fibre conformation (Fig. 5; ESM Fig. S3). UV radiation causes a marked increase in crystallinity, which tends to stabilise after prolonged exposure. Thermo-oxidation at 125 °C also seems to cause a subtle increase in the crystallinity index, but far less obvious than UV radiation. Finally, pH exposure at extremely acidic and alkaline conditions (pH 1 and pH 13, respectively) also seems to increase the crystallinity index. Exposure to different RH conditions does not seem to alter the conformation at the amide III region. In the case of the historic samples, the crystalline index for the GIIA doublet shows a slight increase in the crystalline index, whereas the remaining samples show similar values as the reference silk. This increase in crystallinity may be caused by chain scission reactions in the amorphous (random coil) regions of the silk fibres leading to fragments of lower molecular weight that can be more easily arranged into crystalline segments. These results point out that the amide III crystalline index constitutes a sensitive marker for monitoring the structural changes in historic silk textiles.

Finally, UV radiation causes a drastic reduction in the intensity of the tyrosine doublet band at wavenumbers of 835 and 850 cm^−1^ (Fig. 4e); this is a consequence of the photo-oxidation of the chromophoric tyrosine amino acids that are present in the amorphous silk regions [23]. The historic samples exhibit a decrease in the tyrosine doublet as well, but not as drastic as the one caused by UV exposure. A similar decrease in the tyrosine bands can be observed for the sample thermo-oxidised at 125 °C for 56 days. These changes in the Tyr intensity are confirmed by the amino acid results (see “Amino acid composition”). This indicates that accelerated UV exposure causes more severe degradation effects than the ones undergone by silk during its natural ageing, indicating that this artificial ageing procedure should be used with caution for mimicking the degradation state of historic textiles.

### Amino acid composition

The results of the amino acid analyses for the selected historic and artificially aged silk samples can be found in Table 1, expressed as the percentage of the total amount of amino acids. In general, there is a fine conformity between the calculated model silk sequence (MSS) and the results, especially with the results of the unaged reference silk. The deviating values of Ser and Glu may be due to the poor separation of the two peaks during chromatography. Gly and Ala constitute the core of the amino acid sequence in the crystalline domains of silk and are usually regarded as stable amino acids. According to the model reference, a small amount of thiopropionyl-cystine (TPCys; which is a marker for cystine and cysteine) should be expected, but it has only been detected in the UV-aged samples. TPCys may be present in all samples but in such small amounts that the TPCys peak is hidden under the delayed Ala peak.

**Table 1:** Amino acid composition (%) of historic and selected artificially aged silk textile samples

Sample	Amino acid composition (%)																		Tyr/Gly^d^	Tyr/Ala	Protein cont. %
	Asp^b^	Thr	Try^c^	Ser	Glu^b^	Pro	Gly	Ala	TPCys	Val	Met	Ile	Leu	Tyr	Phe	His	Lys	Arg			
MSS^a^	1.51	0.98	0.24	11.97	1.11	0.43	44.14	29.44	0.14	2.05	0.08	0.62	0.46	5.19	0.68	0.21	0.29	0.46	0.12	0.18	–
Reference silk	1.51 (0.01)	1.05 (0.21)	–	10.28 (0.15)	1.29 (0.00)	0.44 (0.01)	44.31 (0.06)	30.96 (0.05)	–	2.05 (0.02)	0.06 (0.00)	0.62 (0.01)	0.45 (0.01)	5.18 (0.00)	0.73 (0.01)	0.28 (0.04)	–	0.37 (0.03)	0.12	0.17	96.7
UV4d	1.59 (0.01)	0.97 (0.07)	–	10.37 (0.10)	1.32 (0.02)	0.44 (0.02)	43.66 (0.64)	32.41 (0.23)	0.45 (0.23)	2.17 (0.06)	0.06 (0.01)	0.59 (0.00)	0.49 (0.01)	3.80 (0.00)	0.71 (0.01)	0.21 (0.07)	–	0.31 (0.05)	0.09	0.12	85.0
UV10d	1.49 (0.05)	1.07 (0.12)	–	10.18 (0.06)	1.23 (0.03)	0.33 (0.06)	44.15 (0.13)	33.06 (0.17)	0.68 (0.23)	2.13 (0.01)	0.05 (0.00)	0.55 (0.00)	0.47 (0.00)	3.08 (0.03)	0.66 (0.01)	0.18 (0.02)	–	0.29 (0.03)	0.07	0.09	80.9
T125C 28d	1.52 (0.02)	0.91 (0.08)	–	10.12 (0.12)	1.30 (0.01)	0.41 (0.03)	44.67 (0.03)	31.52 (0.03)	–	2.10 (0.04)	0.04 (0.00)	0.59 (0.01)	0.49 (0.01)	4.70 (0.04)	0.73 (0.02)	0.23 (0.00)	–	0.26 (0.03)	0.11	0.15	87.7
T125C 56d	1.68 (0.01)	0.67 (0.02)	–	9.25 (0.03)	1.38 (0.02)	0.42 (0.02)	43.87 (0.05)	33.09 (0.12)	–	2.18 (0.01)	0.02 (0.01)	0.62 (0.01)	0.54 (0.02)	4.32 (0.04)	0.72 (0.05)	0.41 (0.05)	–	0.37 (0.02)	0.10	0.13	90.0
RH100 28d	1.59 (0.00)	0.71 (0.01)	–	9.81 (0.04)	1.33 (0.01)	0.45 (0.02)	43.94 (0.10)	31.28 (0.13)	–	2.23 (0.03)	0.06 (0.00)	0.63 (0.02)	0.52 (0.01)	5.24 (0.02)	0.75 (0.00)	0.46 (0.01)	–	0.50 (0.01)	0.12	0.17	95.0
pH1 28d	1.51 (0.06)	0.72 (0.01)	–	10.07 (0.14)	1.37 (0.03)	0.45 (0.06)	42.23 (0.74)	32.46 (0.36)	–	2.19 (0.05)	0.07 (0.00)	0.64 (0.01)	0.50 (0.02)	5.55 (0.11)	0.80 (0.02)	0.46 (0.06)	–	0.51 (0.06)	0.13	0.17	93.4
pH13 28d	0.86 (0.01)	0.47 (0.04)	–	9.84 (0.52)	1.01 (0.17)	0.37 (0.08)	43.75 (1.04)	33.96 (0.45)	–	1.95 (0.01)	0.03 (0.00)	0.38 (0.00)	0.27 (0.01)	5.38 (0.01)	0.55 (0.01)	0.47 (0.07)	–	0.37 (0.04)	0.12	0.16	97.3
GIIA doublet	1.63 (0.09)	0.97 (0.07)	–	10.61 (0.05)	1.40 (0.04)	0.47 (0.06)	43.86 (1.15)	31.00 (0.76)	–	2.21 (0.04)	0.06 (0.02)	0.62 (0.02)	0.52 (0.00)	4.81 (0.46)	0.77 (0.02)	0.28 (0.02)	–	0.33 (0.04)	0.11	0.16	76.2
GIIA breeches	1.61 (0.02)	1.13 (0.08)	–	10.34 (0.12)	1.36 (0.01)	0.45 (0.00)	43.90 (0.07)	31.66 (0.02)	–	2.10 (0.03)	0.05 (0.00)	0.61 (0.00)	0.47 (0.01)	4.62 (0.01)	0.72 (0.01)	0.26 (0.01)	–	0.29 (0.01)	0.11	0.15	86.0
KXG cloak	1.62 (0.01)	1.11 (0.13)	–	10.28 (0.16)	1.41 (0.01)	0.53 (0.02)	43.74 (0.87)	31.50 (0.73)	–	2.14 (0.01)	0.06 (0.00)	0.61 (0.01)	0.47 (0.01)	4.78 (0.06)	0.75 (0.03)	0.29 (0.01)	–	0.30 (0.05)	0.11	0.15	83.2

The standard deviation (SD) is presented between brackets

^a^The model silk sequence (MSS) is calculated from the amino acid sequences of the *Bombyx mori* Fibroin heavy chain (sequence number P05790), *B. mori* Fibroin light chain (sequence number P21828) as well as *B. mori* Fibroin P25 (sequence number P04148), where the reported values are an addition between the three in a ratio 6:6:1, respectively

^b^The value of Glu is representing both glutamin and glutamic acid, and Asp is representing both asparagin and aspartic acid

^c^Tryptophan is only present in the model reference as it is destroyed during the acid hydrolysis of the samples

^d^Tyr/Gly ratio according to Zhang et al. [23]

Tyrosine content (Tyr) is known as a marker for oxidation in silk fibroin [9]. The unaged reference silk shows a Tyr value very similar to the model reference, which again indicates the robustness of the amino acid quantification. Accelerated UV exposure causes a marked decrease of the Tyr value, which almost decreases by half after 10 days of exposure. Thermo-oxidation also causes a progressive decrease in the amount of Tyr, but this is moderate in comparison with UV exposure. On the other hand, no decrease of Tyr can be observed in the samples treated neither at pH 1 and pH 13 nor with 100 % RH exposure. The historic samples also exhibit lower Tyr values, which again indicate that they have been subjected to some degree of oxidation during their natural ageing. The low Tyr values in the historic and the artificially aged (UV and thermo-oxidised) silk textiles may indicate the occurrence of oxidative reactions in the amorphous spacers within the silk structure, in agreement with the FTIR results. Indeed, it is known that the amorphous spacers are rich in Tyr that will be more susceptible to oxidative reactions than the crystalline regions. In the case of the UV and thermo-oxidised samples, the results point towards to the transformation of Tyr into Ala by loss of the phenol group [9, 24] and the formation of oxidative derivatives such as hydroxyphenylalanine and quinones [25, 26]. Zhang et al. [24] use the molar ratio Tyr/Gly to indicate presence of sericin, but due to tyrosine’s sensitivity towards oxidation the ratio may also be used as an indicator of changes within the silk fibroin structure. The Tyr/Gly ratios are presented in Table 1, which show a decrease for the samples exposed to UV compared with the reference and historic silk textiles. An even better marker for increasing oxidative changes may be found in the decreasing Tyr/Ala ratio (Table 1), where Tyr in many cases is transformed into Ala. Again, it can be observed that the UV exposure surpasses the effect caused by thermo-oxidation in comparison with the historic costumes. No appreciable decrease in the Tyr content is observed for the samples exposed to RH and immersed into extreme acidic and alkaline pH conditions. These results point out that Tyr oxidation is negligible in these exposure conditions.

Immersion into extreme acidic and alkaline pH environments (pH 1 and pH 13, respectively) is expected to cause hydrolysis and chain scission of the peptide bonds in silk fibroin. This hydrolysis is expected to occur predominantly in the amorphous spacers within the fibroin chains, which are in principle more sensitive to degradative effects compared with the crystalline regions. Indeed, a meaningful reduction in the amino acids with polar side chains (Asp, Thr, Ser) and with hydrophobic side chains (Ile, Leu) is observed, especially for the sample subjected to pH 13. These amino acids are particularly found in the amorphous regions connected to the Gly-X crystalline domains, which indicates the special sensitivity of these amorphous spacers to alkaline hydrolysis. A progressive decrease in amino acids with polar chains (especially Ser and Thr, but not Asp) is similarly observed for the samples exposed to thermo-oxidation.

Traces of the breakdown product γ-Abu are found in some historic samples (KXG cloak and GIIA doublet) and in the samples exposed to UV and thermo-oxidation at 125 °C (data not shown). γ-Abu is most likely originating from Pro and Lys [27]. All samples show a small peak right before Lys. This peak is identical with Ornithin, a breakdown product originating from Arg. None of the samples showed traces of the degradation products Ada, Abu, β-Ala and 6-Aha.

From the amino acid analysis, it can be concluded that environmental degradation causes important changes in the molecular structure of silk fibroin, affecting predominantly the amino acids contained in the amorphous regions. UV and thermo-oxidation seem to cause the oxidation of the Tyr group and an increase in the formation of Ala, which may lead to an increase of the crystallinity and changes in the mechanical performance. A progressive loss of Ser is also observed in these samples, which may affect the crystallinity of these samples. On the other hand, severe acid and alkaline conditions seem not to affect Tyr but cause hydrolysis in the amino acids containing polar and bulky side chains in the amorphous regions, which seem to be the weakest points for hydrolysis in the silk structure.

### Size-exclusion chromatography

**Fig. 6:**
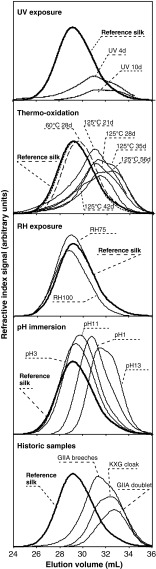
SEC chromatograms for the artificially aged and historic silk samples: **a** UV exposure; **b** thermo-oxidation; **c** RH exposure at 25 °C for 28 days; **d** immersion into pH solutions at 25 °C for 28 days; and **e** historic samples

The relative molecular weight distributions of the artificially aged and historic silk samples were evaluated with SEC in *N*,*N*-dimethylacetamide (DMAc)/0.5 % (*w*/*w*) LiCl as the solvent system. Reference silk presents a monomodal SEC distribution (Fig. 6) with large (poly)dispersity, which is expected to cover both populations of H- and L-fibroins. Exposure to UV irradiation causes a shift in the retention time of the SEC chromatogram to higher elution volumes and the formation of a bimodal distribution, evidencing the reduction in chain length caused by UV degradation. It is worth mentioning that the presence of insoluble particles was observed in the DMAc/LiCl solutions of UV-irradiated silk; these particles were filtered prior to SEC analysis to avoid column deterioration and were therefore not analysed. The formation of insoluble particles was not reported neither for other degraded samples in this study nor for the historic silk samples. The formation of these particles may indicate the occurrence of crosslinking caused by UV irradiation in addition to oxidation and chain scission, as it has been reported for other macromolecules [28].

Exposure to thermo-oxidation at moderate temperatures (up to 60 °C) does not seem to affect silk chain length after 28 days. However, exposure to high temperatures (125 °C) causes a progressive shift of the chromatograms to higher elution volumes. The formation of bimodal distributions is observed as well for the thermo-oxidised samples at 125 °C, indicating the formation of two macromolecular populations of different chain lengths by chain scission. The relative abundance of these macromolecular population changes progressively with exposure times as it can be observed by the relative peak heights in the SEC chromatograms, with increasing relative abundance of the population of lower chain lengths at higher elution volumes with increasing exposure.

Exposure to relative humidity does not affect the shapes of the SEC distributions, which indicates that high RH values at moderate temperatures do not induce chain scission reactions in the time frames of the present study. Immersion into extreme pH solutions at 25 °C, on the other hand, causes a shift of the SEC distributions to higher elution volumes, evidencing the effect of hydrolysis of the peptide chains. This effect is more marked for extreme alkaline conditions (pH 13), which effectively causes extensive hydrolysis and the formation of the degraded bimodal distribution already observed for the UV and thermo-oxidised samples at 125 °C.

The historic samples all exhibit similar SEC bimodal distributions with lower chain length as the reference silk, evidencing the occurrence of chain scission of the peptide silk chains. This reduction of molar mass is indeed a common feature in organic archaeological materials and artefacts. The degree of degradation can therefore be monitored for the historic samples by the relative abundance of such macromolecular populations with reduced chain length. From the results of the SEC analyses, it seems that thermo-oxidation at 125 °C using different degradation times can be effectively used to model the chain length distribution of historic samples subjected to different degrees of degradation.

### Tensile properties

To validate the four methods for artificial ageing against the properties of seventeenth-century silk fabric, tensile tests were used to compare the mechanical markers of historic silk samples with those of artificially aged samples (Table 2). The tenacity indicates the resistance to steady forces and will be the suitable magnitude to consider when a specimen is subjected to a steady pull. Elongation at break and tenacity should be regarded as the most important mechanical properties in conservation science when comparing artificially aged samples with historic silk. These properties are relevant when a specimen is subjected to stretching, as when the neck of a garment is being pulled over the head and when costumes are being mounted on mannequins during museum handling [29].

**Table 2:** Mechanical properties of historic and artificially aged silk textile samples.

			Modulus (cN/tex)	Elongation at break (%)	Tenacity at break (cN/tex)
Unaged reference silk					
Unaged silk			191.5 (44.4)	21.0 (4.4)	31.8 (4.7)
Historic silk					
GIIA doublet			304.0 (66.8)	2.2 (0.8)	5.8 (2.2)
GIIA breeches			240.0 (61.8)	2.7 (0.6)	4.8 (2.7)
KXG cloak			274.0 (29.3)	3.3 (0.3)	5.9 (0.9)
UV exposure					
Temperature (°C)	Time (days)	RH (%)	Modulus (cN/tex)	Elongation at break (%)	Tenacity at break (cN/tex)
50 ± 2 °C	1	95	337.0 (33.9)**	11.2 (1.8)**	17.6 (3.6)
	2	95	267.0 (14.4)***	8.9 (0.4)***	13.7 (0.8)***
	3	95	266.4 (6.4)***	7.2 (0.3)***	10.6 (0.4)***
	4	95	226.0 (14.8)	5.4 (0.2)***	6.2 (0.5)***
	5	95	189.8 (27.2)	5.3 (0.4)***	5.8 (0.5)***
	6	95	126.0 (8.9)***	4.3 (0.6)***	4.0 (0.7)***
	7	95	109.2 (15.8)***	4.5 (0.4)***	3.7 (0.7)***
	8	95	103.0 (7.5)***	3.8 (0.2)***	2.5 (0.4)***
	9	95	70.8 (8.3)***	3.4 (0.2)***	2.1 (0.2)***
	10	95	62.0 (1.7)***	2.6 (0.5)***	1.5 (0.1)***
Thermo-oxidative exposure					
Temperature (°C)	Time (days)	RH (%)	Modulus (cN/tex)	Elongation at break (%)	Tenacity at break (cN/tex)
25	28	53	233.0 (41.1)	25.4 (5.6)	30.1 (5.2)
60	28	53	230.0 (47.8)	18.4 (3.9)	28.8 (6.6)
125	14	0	296.0 (8.1)***	9.0 (0.1)***	15.0 (0.2)***
	21	0	252.0 (15.8)	9.7 (0.3)***	14.7 (1.1)***
	28	0	208.0 (11.4)***	5.6 (0.3)***	7.2 (0.2)***
	35	0	132.0 (16.7)***	5.5 (0.2)***	4.3 (0.3)***
	42	0	119.0 (13.3)*	4.6 (0.1)***	3.1 (0.5)***
	49	0	143.0 (44.4)*	5.1 (0.3)***	4.3 (0.4)***
	56	0	106.0 (8.6)**	5.1 (0.5)***	3.3 (0.5)***
Humidity exposure					
Temperature (°C)	Time (days)	RH (%)	Modulus (cN/tex)	Elongation at break (%)	Tenacity at break (cN/tex)
25	28	0	194.0 (26.1)	20.5 (3.6)	23.0 (3.7)
		53	233.0 (41.1)	23.6 (3.9)	30.1 (5.2)
		75	231.0 (18.0)	20.7 (4.6)	34.1 (7.0)
		86	229.0 (25.4)	23.7 (1.2)	32.1 (7.6)
		100	211.0 (20.0)	22.3 (4.0)	30.2 (2.0)
60	28	0	224.0 (12.0)	17.7 (4.9)	27.5 (3.8)
		53	230.0 (47.8)	18.4 (3.9)	28.8 (6.6)
		75	219.0 (5.7)	20.8 (8.2)	29.9 (5.9)
		86	232.0 (12.4)	21.4 (1.1)	31.0 (4.8)
		100	175.0 (11.4)	20.6 (2.1)	23.4 (3.0)*
125	28	0	208.0 (11.4)	9.0 (0.1)***	15.0 (0.2)***
pH immersion					
Temperature (°C)	Time (days)	pH	Modulus (cN/tex)	Elongation at break (%)	Tenacity at break (cN/tex)
25	28	1	191.0 (11.2)	16.8 (1.3)	24.1 (6.6)***
		3	209.0 (6.9)*	22.8 (0.8)*	25.0 (4.0)
		7	198.0 (30.8)	19.5 (3.1)	30.0 (2.9)
		11	202.0 (6.9)	19.8 (2.8)	29.8 (2.3)
		13	–	–	–
60	28	1	–	–	–
		3	253.0 (12.3)	16.6 (1.6)	28.6 (3.8)
		7	215.0 (14.8)	23.7 (1.0)	32.6 (3.0)
		11	224.0 (6.1)	15.7 (1.7)	25.1 (2.4)
		13	–	–	–

The figures between brackets are standard deviation

*p < 0.05; **p < 0.01; ***p = 0.000. Asterisks denote significant differences from unaged reference silk

*Accelerated ageing by UV exposure* results in a sudden increase of the modulus after short exposure times (1–3 days) compared with the reference silk and a progressive decrease thereafter. On the other hand, the values of tenacity and elongation at break decrease progressively when compared with the reference silk. This sudden increase of the elastic modulus after only one day of UV irradiation may be explained by the physical reorganisation of the silk chains in the amorphous regions at moderate temperatures above room temperature, a typical phenomenon known as physical ageing in polymer science [30]. After this initial exposure, chemical changes caused by UV radiation become irreversible as shown by FTIR, SEC and amino acid composition, which results in a progressive deterioration of the mechanical properties. This indicates that degradation by UV irradiation does not correspond appropriately with the natural degradation undergone by the historic silk.

*Thermo-oxidative exposure at higher temperatures* (125 °C) reduces break extension and tenacity already from the shortest time exposure with some levelling effects after 28 days. The results for modulus were however different. The modulus after thermo-oxidative exposures at short times was significantly higher than the modulus of unaged reference silk and was not significantly reduced until 42 days exposure. This could be related to physical chain relaxation effects of the silk chains during initial thermo-oxidative exposure, as it is also seen for the samples exposed at lower temperatures (60 °C).

*Relative humidity (RH)* seems to have very little effect on the mechanical properties of accelerated aged silk. When the two sources of variance, humidity (0, 53, 75, 86, 100 %) and temperature (25, 60, 125 °C), were analysed separately in univariate analysis of variance, only one difference in break extension was significant: 0 % RH in 125 °C (*p* = 0.016). It is therefore the exposure to heat that causes the differences. The three temperatures explain more variance in break extension than the five humidity levels (partial eta squared equals 0.88 and 0.55, respectively). However, as the values demonstrate in Table 2, it is the exposure at 125 °C that has the decisive effect. In modulus, there are no significant differences between exposed and unexposed silk. In tenacity, significant reductions were seen at RH 100 % at 60 °C and at RH 0 % at 125 °C, and in break extension, a significant reduction was only obtained at RH 0 % at 125 °C.

*Immersion of the silk samples in extreme pH conditions* caused severe effects on their mechanical properties. The exposure at pH 13 at 25 and 60 °C and at pH 1 at 60 °C had such a deleterious effect that the silk could not be tested mechanically. In the remaining exposure conditions, pH 1, 3, 7 and 11 in 25 °C and pH 3, 7 and 11 in 60 °C (Table 2), Young’s modulus was not at all significantly affected, and tenacity decreased significantly only after exposure to pH 1 at 25 °C. Break extension was significantly reduced at pH 1 at 25 °C and at 60 °C at pH 1 and pH 13. However, the levels of extension and tenacity even after prolonged exposures were much higher than the historic silk.

*Historic silk samples* show slightly higher modulus values and much-reduced elongation at break and tenacity in comparison with the reference silk. Indeed, the seventeenth-century silk shows on average a tenacity that is 17 % of the reference silk. This is a clear indication of the brittleness of the historic silk fibres caused by combined physical (e.g. chain reorganisation) and chemical (e.g. chain scission and oxidation reactions) structural effects during their natural ageing.

The mechanical results when using thermo-oxidative exposure are fairly similar to the fragile historic silk, especially when balancing the high elastic modulus and the reduced elongation at break and tenacity. The reductions in elongation at break and tenacity reach the low level of historic silk when aged for 4 days or more. It seems that competitive or combined physical and chemical degradation occurred in historic silk, leading to such mechanical properties that are difficult to model using accelerated ageing tests. However, thermo-oxidative ageing at 125 °C allows a certain control of the divergent effects on the elastic modulus and the elongation at break by selecting the exposure times, which may be related to the predominance of physical and irreversible chemical changes. We have already emphasised that break extension and tenacity are the most important properties to achieve for conservation purposes and must have precedence over the elastic modulus. The discrepancy in the modulus values between the historic samples and the artificial aged samples might depend on the sericin remains and the resinous traces found. Therefore, according to the tensile tests thermo-oxidation is the artificial ageing method that gives silk properties closest to the historic silk.

### Integration of the analytical results using exploratory principal component analysis

**Fig. 7:**
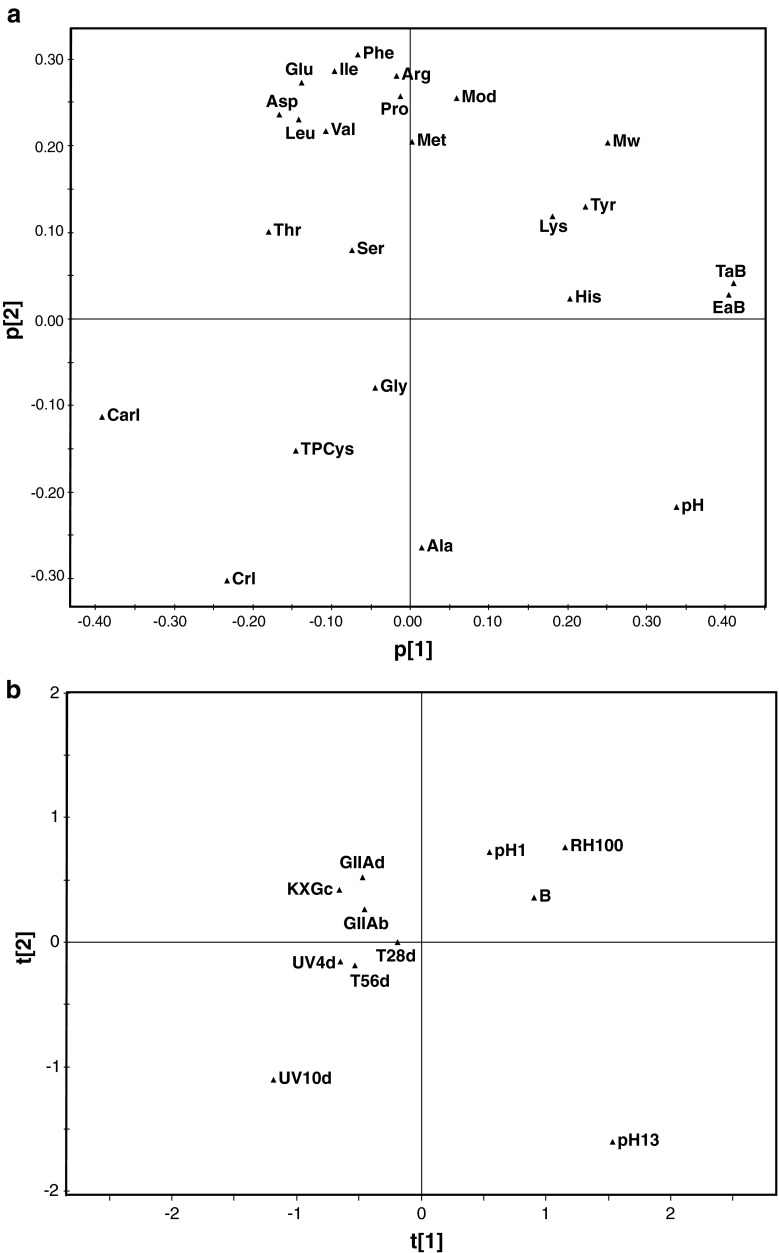
**a** Exploratory principal component analysis (PCA) of the analytical markers for the degradation of historic and artificially aged silk textiles. Loading plot for the analytical markers (variables) under the first two principal components. These two principal components explain 66.8 % of the total variance. Nomenclature: amino acid composition (as in Table 1), carbonyl index (*CarbI*), crystalline index (*CrI*), molar mass from SEC (*Mw*), acidity (*pH*), Young’s modulus from tensile tests (*Mod*), elongation at break (*EaB*), tenacity at break (*TaB*); **b** PCA score plot for the silk textile samples (observations) under the first two principal components. Nomenclature: *B* reference silk, *UV4d* UV irradiation (4 days), *UV10d* UV irradiation (10 days), *T28d* thermo-oxidation (125 °C, 28 days); King Gustav II Adolf doublet from 1617, King Gustav II Adolf breeches from 1617 and King Karl X Gustav cloak from 1654

The analytical markers monitoring the structure and properties of silk were statistically integrated using exploratory principal component analysis (PCA), to obtain correlations between the effect of the different artificial ageing environments and the properties of the historic silk samples. Only 11 artificially aged and historical silk samples were available for the statistical analyses; in them, all the qualitative markers were accessible. These observations include the reference silk, UV irradiation for 4 days, UV irradiation for 10 days, thermo-oxidation at 125 °C for 28 days, thermo-oxidation at 125 °C for 56 days, immersion at pH 1 at 25 °C for 28 days, immersion at pH 13 at 25 °C for 28 days, exposure at 100 % RH at 25 °C for 28 days, King Gustav II Adolf’s doublet from 1617, King Gustav II Adolf’s breeches from 1617 and King Karl X Gustav’s cloak from 1654. The variables (analytical markers) comprised the amino acid composition, carbonyl index, crystalline index, molar mass from SEC, acidity, Young’s modulus, elongation at break and tenacity at break (see Electronic Supplementary Material). Taking into account these results, we decided to implement the exploratory PCA model using a total of 11 samples (observations) and 24 variables (analytical markers), as described in Fig. 7. We are aware of the limitations imposed by the few number of observations related to the large number of variables, which restrict the significance of the multivariate analysis. However, we believe that the exploratory PCA plots may be very useful to integrate the information from the analytical markers of silk degradation and to offer indicative correlations amongst the historic silk textiles and silk samples artificially aged in different environments. The PCA model was created employing SIMCA-P package from Umetrics (Sweden) using the correlation matrix. Four principal components with Eigenvalues larger than unity were derived from the analysis; principal components PC1 to PC4 explained 38.9, 27.9, 11.9 and 9.6 % of the variance in the data set, respectively, and 88.3 % of the total variance altogether. The loading matrix for the different principal components is presented in the ESM Table S1.

Figure 7a shows the principal component loading plot for the different analytical markers distributed in the surface according to their contribution to the two main principal components (PC1 and PC2). Together, these two principal components explain 66.8 % of the total variance. The different amino acid components appear clustered according to their sensitivity towards degradation; the placement of Ser and Thr clustered together is noteworthy, compared with Tyr, Lys and His on another side, and finally, Ala on opposite sides. This distribution of the amino acids in the loading plot correlates well with the effect of the different degradation mechanisms (oxidation and hydrolysis) on the amino acid composition. Similarly, the carbonyl index (CarbI) from FTIR and the tyrosine content (Tyr) are inversely placed on the loading plot, evidencing the direct connection between the decrease of the Tyr content by oxidative reduction and the increase of the carbonyl groups, which may be related to the formation of quinones [25, 26] as discussed in “Amino acid composition.” The reciprocal placement of the molecular weight (from SEC) and the crystalline index (from FTIR) may be related to the occurrence of hydrolysis during silk degradation. Indeed, a decrease in the molar mass (from SEC) caused by chain scission of the amorphous regions leads to shorter fibroin chains that may reorganise in crystalline structures, therefore causing an increase in the crystallinity index (from FTIR). This phenomenon is commonly observed in the degradation of synthetic and natural semi crystalline polymers. Finally, the decrease in the mechanical properties (mainly elongation and tenacity at break) correlates well with structural markers such as the decrease of the Tyr content and the molar mass, and the increase of the carbonyl and the crystalline indexes. The alteration of the organisation of silk fibroin at the primary (amino acid composition) and secondary (crystallinity) structural levels and the formation of oxidative moieties (e.g. carbonyl groups) may induce an overall weakening of the structural integrity of the silk fibres. However, these structural changes induced by competitive or combined physical and chemical degradation mechanisms, lead to interconnected effects on the modulus, elongation and tenacity at break that are difficult to control by accelerated ageing procedures, as stated in “Tensile properties.”

Exploratory PCA provides as well interesting information of the effects of the different degradation environments on the properties of silk compared with historic samples in the principal component scores plot of the observations projected on a model hyperplane (Fig. 7b). The samples subjected to 100 % relative humidity (RH100) and extreme pH environments (pH 1 and pH 13) appear dispersed and far away from the historic samples. It is apparent that exposure to RH and immersion into extreme acidic and alkaline pH environments do not induce similar degradation effects that historic silk textiles have undergone. Exposure to RH and immersion in acidic pH are consistently grouped together with the reference silk sample based on the analytical markers; this indicates that these degradation environments do not alter to a great extent the structure and properties of silk textiles. Immersion into extreme alkaline pH solutions, however, seems to cause severe hydrolysis of the peptide chains to an extent that the fibrilar configuration is disrupted and the mechanical properties are lost. On the other hand, thermo-oxidation at high temperatures (125 °C) and UV exposure seem to reproduce in a closer way the analytical markers of historic silk, especially at shorter exposure times. This seems to be caused by a combination of oxidation, chain scission (hydrolysis) and physical processes that occur synergistically to different extents in the artificially aged samples exposed to UV, thermo-oxidation and in the historic samples. However, prolonged UV exposure seems to induce harsher and divergent degradation effects compared with the historic samples, as indicated by the complete reduction of the Tyr groups (see “Fourier-transform infrared spectroscopy” and “Amino acid composition”) and the occurrence of crosslinking (as mentioned in “Size-exclusion chromatography”). From these results, it can be concluded that thermo-oxidative exposure at 125 °C provides artificially aged silk samples with the properties closest to those of historic textiles, and that it is possible to mimic the degree of degradation of the historic silk samples by tailoring the exposure time to thermo-oxidation.

## Conclusions

The preservation of our historic heritage requires the multidisciplinary cooperation of experts in art history and conservation science, materials science and analytical chemistry. In this work, suitable analytical markers have been identified to monitor the degree of degradation of historic silk textiles based on the changes in the chemical structure and in the macroscopic properties. Integration of these analytical results is crucial to establish correlations amongst properties but also to obtain fundamental knowledge of the mechanisms of deterioration caused by different environments. Accelerated ageing methods are useful to understand the degradation mechanisms to which historic samples are subjected, but their influence must be considered with precaution. This study proved that thermo-oxidation at elevated temperatures (here 125 °C) is the accelerated ageing procedure that best mimics the degradation state of historic silk textiles, and that the degree of degradation can be controlled by the exposure time. Oxidation, hydrolysis, chain scission and chain rearrangements (physical ageing) are shown to be the main degradation mechanisms affecting the structure and properties of silk textiles. The integration of accelerated ageing procedures with the identification of analytical markers proves to be a valuable procedure to support the conservation tasks currently performed in our museums and as a starting point for large-scale assessment of the degree of degradation of silk textiles from different historic periods. In addition to this, it provides the basic scientific knowledge for studying the in vitro and in vivo degradation of silk-based biomaterials, with significance in biomedical applications.

## Electronic supplementary material

Below is the link to the electronic supplementary material.

ESM 1 (PDF 95 kb)
