# Supplementary material for: Analytical markers for silk degradation: comparing historic silk and silk artificially aged in different environments
Source: Anal Bioanal Chem. 2014 Dec 10;407(5):1433–49. doi: 10.1007/s00216-014-8361-z (PMC4318988; doi:10.1007/s00216-014-8361-z)
Supplement: Supplementary file 1 — (PDF 95 kb) [file 216_2014_8361_MOESM1_ESM.pdf]

Analytical and Bioanalytical Chemistry

Electronic Supplementary Material

Analytical markers for silk degradation: Comparing historic silk and silk artificially aged in different environments

Francisco Vilaplana, Johanna Nilsson, Dorte Vestergaard Poulsen Sommer,
Sigbritt Karlsson

Brightness measurements

RH exposure at 25°C does not induce significant brightness changes after 28 days of exposure, similarly to what was observed for RH exposure at 60°C (Fig. 3). In the same direction, pH exposure at neither 25°C nor 60°C for 28 days induces significant changes in brightness. This observation is opposed to the marked decrease in brightness for the silk samples immersed for 28 days in extreme pH conditions (both acidic and alkaline) observed in Fig. 3.

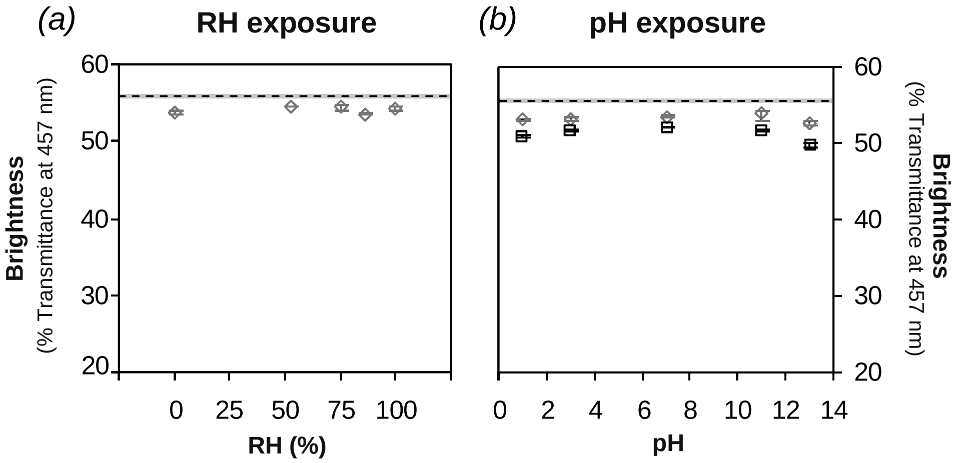

Fig. S1 Effect of the different artificial degradation environments on the brightness (expressed as % of transmittance at 457 nm) of silk textiles. (a) RH exposure (25°C). (b) pH exposure (empty black square: 60°C; empty grey rhombus: 25°C). The area in grey corresponds with the reference silk

Selection of the Amide I peak as reference peak for carbonyl index.

An interesting feature of this study is that neither artificial nor natural ageing affect to a great extent the features of the main peak corresponding to Amide I (Fig. 4c), even if silk hydrolysis is observed in some cases after degradative exposure. In order to verify the viability of the Amide I peak as a reference for the calculation of the carbonyl index, the heights of the Amide I peak after baseline correction and spectral normalization (see section 2.5) have been plotted for all the samples (Fig. S2). The Amide I peak heights are basically not affected neither in any of the artificial degradation environments nor in the historic samples, in comparison with the artificial silk. This proves that the selection of the Amide I peak for the calculation of the carbonyl index is valid for our conditions.

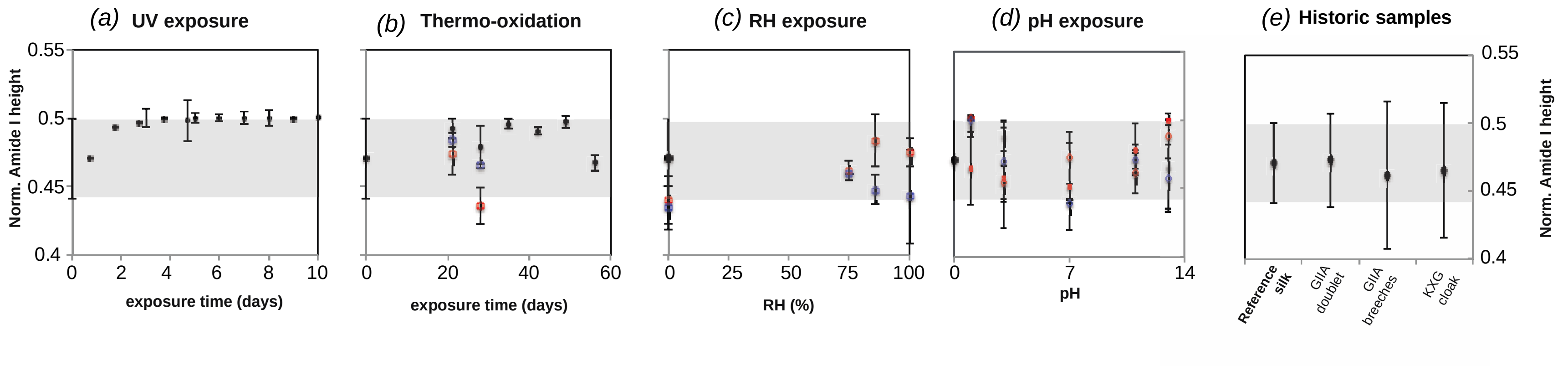

Fig. S2 Variation of the normalized Amide I FTIR peak for the artificially-aged and historic silk samples. (a) UV exposure; (b) Thermo-oxidation (black: 125°C, red: 60°C, blue: 25°C); (c) RH exposure (red: 60°C, blue: 25°C); (d) pH exposure (red: 60°C, blue: 25°C, full squares: immersed, empty rhombus: exposed); (e) historic silk samples. The area in grey corresponds with the variance of the Amide I peak height for the reference silk sample

Carbonyl and crystalline indexes from FTIR

Exposure to environments with different relative humidity (RH) at 25°C does not induce significant changes in neither the carbonyl nor the Amide III crystalline index after 28 days, similarly to what we observed at 60°C (Fig. 5). Exposure to aqueous solutions with different pH induces only a slight increase in the carbonyl index for extreme acidic conditions (pH1 and pH3) at 60°C, which is related to the catalytic activity of temperature to induce hydrolytic reactions on silk fibroin. However, this effect is more limited than the one observed for direct immersion of silk into aqueous solutions with acidic pH (Fig. 5).

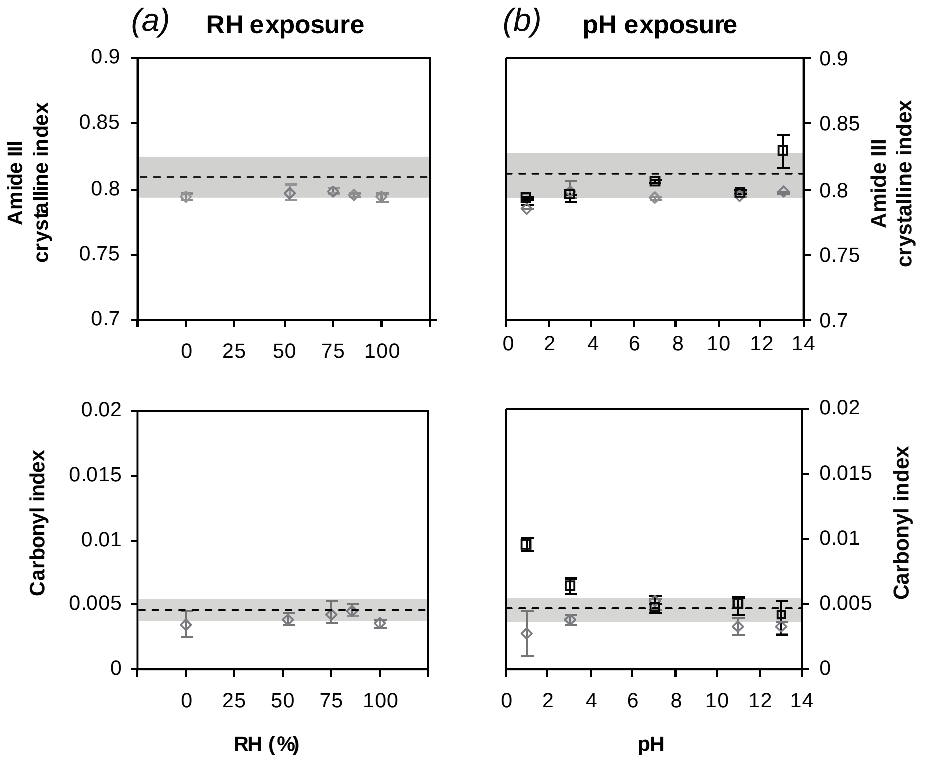

Fig. S3 FTIR crystalline and carbonyl indexes: (a) RH exposure (grey 25°C); (b) pH exposure (black squares: 60°C; grey rhombus: 25°C). The area in grey corresponds with the reference silk

Statistical analysis of the analytical markers for silk degradation

The effect of the different artificial ageing environments on the structure and properties of silk was further studied using different multivariate data analyses. Two independent data pretreatment approaches were performed of the tensile test results and of the amino acid composition; Pearson r correlations between variables were thus assessed for both cases (SPSS Statistics 19 and OriginPro 8.5, respectively). For the tensile tests, a very high correlation between elongation at break and tenacity was observed (r = 0.91) when calculated on all 191 available data; the modulus, on the other hand, exhibits lower correlations with elongation at break and tenacity (0.31 and 0.43, respectively). For the amino acid composition, Pearson tests pointed towards significant correlations (r > 0.60) between all the amino acid contents except for Gly.

Only 11 artificially-aged and historic silk samples were available for the statistical analyses; in them all the qualitative markers were accessible. These observations

include: reference silk (B), UV irradiation for 4 days (UV4d), UV irradiation for 10 days (UV10d), thermo-oxidation at 125°C for 28 days (T28d), thermo-oxidation at 125°C for 56 days (T56d), King Gustav II Adolf's doublet (GIIAd), Gustav II Adolf's breeches (GIIAb), and King Karl X Gustav's cloak (KXGc). The variables comprised amino acid composition (labeled as in Table 1), carbonyl index (CarbI), crystalline index (CrI), molar mass from SEC (Mw), acidity (pH), Young's modulus (Mod), elongation at break (EaB), and tenacity at break (TaB). Exploratory principal component analysis (PCA) was performed using SIMCA-P (Umetrics, Sweden) using the correlation matrix. Block scaling of the variables in the data set was performed to the total variance; the amino acid compositions were scaled together in one block and separately from the other variables to account for unit differences. The analysis rendered four principal components with Eigenvalues > 1 that altogether explain 88.3% of the variance.

Table S1 Principal component loadings (*p*) and communalities (h^2) for the analytical markers of silk degradation after exploratory principal component analysis. For each principal component, the variables with the highest absolute values are marked in bold

Variables	Principal Components				Communality h^2
	PC1	PC2	PC3	PC4	
pH	**0.3383**	-0.2166	0.1826	0.1297	0.2115
Mw	**0.2508**	0.2035	-0.1599	**0.5342**	0.4152
Asp	-0.1667	**0.2358**	-0.1130	0.0632	0.1002
Thr	-0.1799	0.1006	0.1739	**0.2608**	0.1408
Ser	-0.0749	0.0799	**0.3507**	0.2273	0.1867
Glu	-0.1383	**0.2734**	-0.0517	-0.0600	0.1002
Pro	-0.0127	**0.2575**	0.1789	-0.1171	0.1122
Gly	-0.0451	-0.0800	0.1238	**0.2550**	0.0888
Ala	0.0139	**-0.2644**	-0.1746	-0.2017	0.1413
TPCys	-0.1458	-0.1517	-0.1229	**0.2865**	0.1414
Val	-0.1073	**0.2169**	-0.1959	-0.0127	0.0971
Met	0.0018	**0.2044**	0.0887	0.2322	0.1036
Ile	-0.0966	**0.2867**	-0.1295	0.0596	0.1119
Leu	-0.1418	**0.2307**	-0.1952	0.0286	0.1122
Tyr	**0.2222**	0.1295	0.1009	-0.1871	0.1113
Phe	-0.0666	**0.3052**	-0.0663	-0.0169	0.1023
His	0.2026	0.0234	-0.1717	**-0.2797**	0.1493
Lys	0.1810	0.1185	**-0.2612**	-0.0651	0.1193
Arg	-0.0174	**0.2811**	-0.1840	0.0494	0.1156
Mod	0.0584	0.2545	**0.6183**	-0.1309	0.4676
EaB	**0.4051**	0.0283	-0.1717	0.1654	0.2217
TaB	**0.4114**	0.0413	-0.0628	0.1643	0.2019
CarbI	**-0.3924**	-0.1131	-0.1281	-0.0353	0.1844
CrI	-0.2335	**-0.3020**	-0.0081	**0.3431**	0.2635
**Eigenvalue**	4.28	3.07	1.31	1.06	**Total R^2 (%)**
**Variance R^2 (%)**	38.9	27.9	11.9	9.6	88.3

Table S1 presents the statistical results of the exploratory PCA for the four principal components. Interestingly, the first component (PC1) explains most of the variance of important properties such as carbonyl index (CrI and CarbI), elongation and tenacity at break (EaB and TaB), pH, and tyrosine content (Tyr). The second component (PC2), on the other hand, includes mainly the amino acids involved in the amorphous spacers in silk. The third component comprises high loadings for Ser content and the elastic modulus (Mod), whereas component PC4 relates inversely the molar mass (Mw) and the crystalline index.

A preliminary cluster analysis using Ward's Method (SPSS 20 for Windows, IBM) was performed in order to identify relatively homogeneous groups of samples (observations) based on the selected analytical markers that were tested (variables). Ward's method proposed 4 clusters with grouped observations as presented in Table S2. Ward's method groups the three historic samples together, while reference silk, RH100, pH1 and pH13 are never combined with the historic samples. Interestingly Ward's Method brings the two shortest time exposures of UV and high temperature together with the historic silk samples, whereas the two extreme exposure times for UV and thermo-oxidation are clustered together.

Table S2 Cluster analysis of the degradation of historic and artificially-aged silk textiles using Ward's Method. A total of 11 samples (observables) were used in the data set. The cluster number corresponding to historic samples is displayed **in bold.** Nomenclature: B (reference silk), UV4d (UV irradiation, 4 days), UV10d (UV irradiation, 10 days), T28d (thermo-oxidation, 125°C, 28 days); thermo-oxidationKing Gustav II Adolf doublet from 1617King Gustav II Adolf breeches from 1617King Karl X Gustav cloak from 1654

Samples	Ward's Method
Reference silk	1
UV4d	**2**
UV10d	3
T28d	**2**
T56d	3
RH100	1
pH1	1
pH13	4
GIIAd	**2**
GIIAb	**2**
KXGc	**2**

These results from Ward's cluster method correlate fairly well with the principal component scores plot obtained by exploratory PCA (Figure S4); the historic samples were clustered with T28d and UV4d, the reference silk was also clustered with pH1 and RH100, and the sample immersed to pH13 was considered an outlier. The main divergence from Ward's method is considering the longest exposure times to both UV and thermo-oxidation (UV10d and T56d) as part of the same cluster, whereas in the scores plot they are not grouped together. In any case, these clustering methods can be of assistance when assessing the degradation effects of accelerated ageing in different environments versus historic samples.

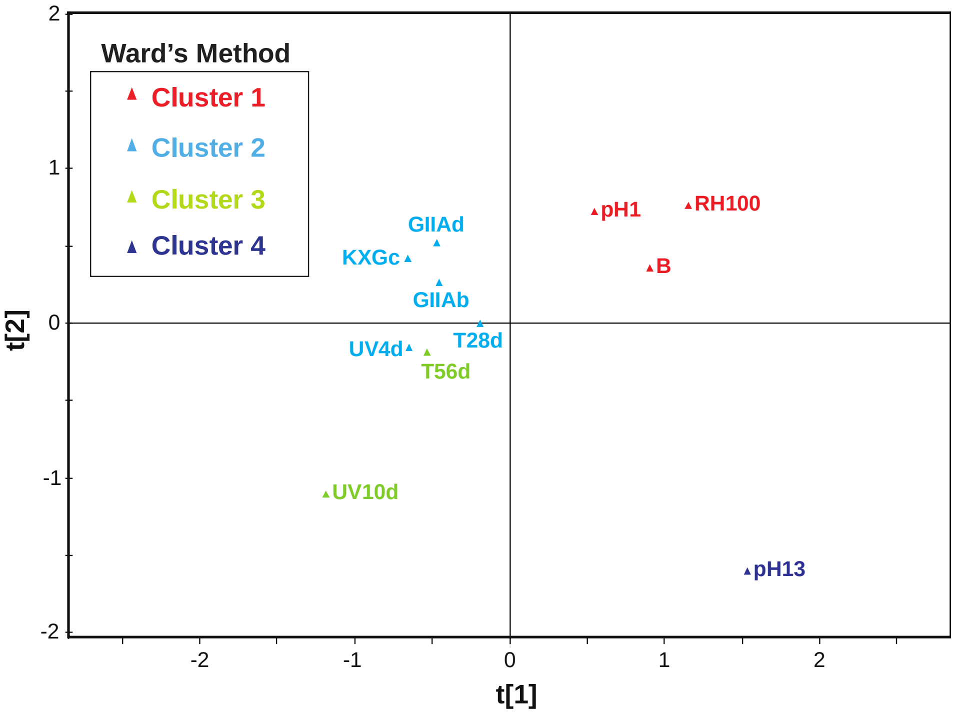

Fig. S4 Comparison of the scores plot of exploratory PCA with the results of Ward's cluster method. Nomenclature: B (reference silk), UV4d (UV irradiation, 4 days), UV10d (UV irradiation, 10 days), T28d (thermo-oxidation, 125°C, 28 days); thermo-oxidationKing Gustav II Adolf doublet from 1617King Gustav II Adolf breeches from 1617King Karl X Gustav cloak from 1654
